# Larval habitat preferences of *Anopheles dirus* and *Anopheles maculatus* in North Sumatra, Indonesia

**DOI:** 10.1186/s13071-026-07441-x

**Published:** 2026-05-18

**Authors:** Boni F. Sebayang, Ahadi Kurniawan, Adzkia M. Haq, Yesika Y. Sianturi, Nursal Nursal, Inke N. D. Lubis, Matthew J. Grigg, Tanya L. Russell, Thomas R. Burkot

**Affiliations:** 1 Australian Institute of Tropical Health and Medicine, James Cook University, Cairns, Australia; 2 Faculty of Medicine, Universitas Sumatera Utara, Medan, Indonesia; 3 Medan Public Health Laboratory Center, Ministry of Health of the Republic of Indonesia, Medan, Indonesia; 4 Faculty of Mathematics and Natural Science, Universitas Sumatera Utara, Medan, Indonesia; 5 Menzies School of Health Research and Charles Darwin University, Darwin, Australia; 6 Institute for Health Research, University of Notre Dame Australia, Fremantle, Australia

**Keywords:** North Sumatra, *Anopheles* larval habitats, *Anopheles maculatus*, *Anopheles dirus*, *Anopheles scanloni*, *Anopheles kochi*, *Anopheles vagus*

## Abstract

**Background:**

While human malaria transmission in Indonesia has declined, reported cases of zoonotic *Plasmodium knowlesi* are increasing. This study examined the larval habitats of malaria vectors in North Sumatra to assess the potential of larval source management as a control strategy for both human and zoonotic malaria vectors.

**Methods:**

Multiple larval habitat surveys in the areas surrounding two dusuns with documented human and zoonotic (*P. knowlesi*) malaria cases in Langkat Regency, North Sumatra, were conducted over 2 years, encompassing both wet and dry seasons. Larval habitats were characterized by mosquito immature presence and density, land-use type where found, aquatic habitat class (i.e., naturally occurring, man-made from natural materials or man-made from artificial materials), and habitat subclass for a range of abiotic and biotic parameters.

**Results:**

A total of 1413 mosquito larvae and 98 pupae were collected. *Anopheles* larvae comprised 20.6% of all mosquito immatures. *Anopheles maculatus* comprised 65.3% of all immature anophelines, followed by *Anopheles dirus* (21.9%), *Anopheles scanloni*, *Anopheles kochi*, and *Anopheles vagus* in order of abundance. Habitat class predicted anopheline presence and density, with larvae occurring more frequently in naturally occurring habitats and man-made habitats from natural materials than in artificial man-made habitats (presence: generalized linear mixed model [GLMM] *β* =  −1.45, *P* = 0.006; density: *β* =  −1.42, *P* = 0.001). At the habitat subclass level, *An. maculatus* larvae were most frequently detected in natural habitats, particularly stream margins. Meanwhile, *An. dirus* larvae occurred approximately equally in natural habitats and man-made habitats from natural materials, with tire tracks being the most common habitat subclass. Four species (*An. maculatus*, *An*. *dirus*, *An*. *scanloni*, and *An*. *kochi*) were detected, albeit infrequently, in man-made habitats from artificial materials.

**Conclusions:**

In North Sumatra, anopheline vector species utilize a diverse range of larval habitats, including natural and man-made, on both natural substrates and artificial materials. Species-specific habitat preferences were observed: *An. dirus* was found in both natural and man-made habitats from natural materials, whereas *An. maculatus* was predominantly found in more dispersed and inaccessible natural sites, suggesting that larval control may be more challenging for *An. maculatus* than *An. dirus*.

**Graphical Abstract:**

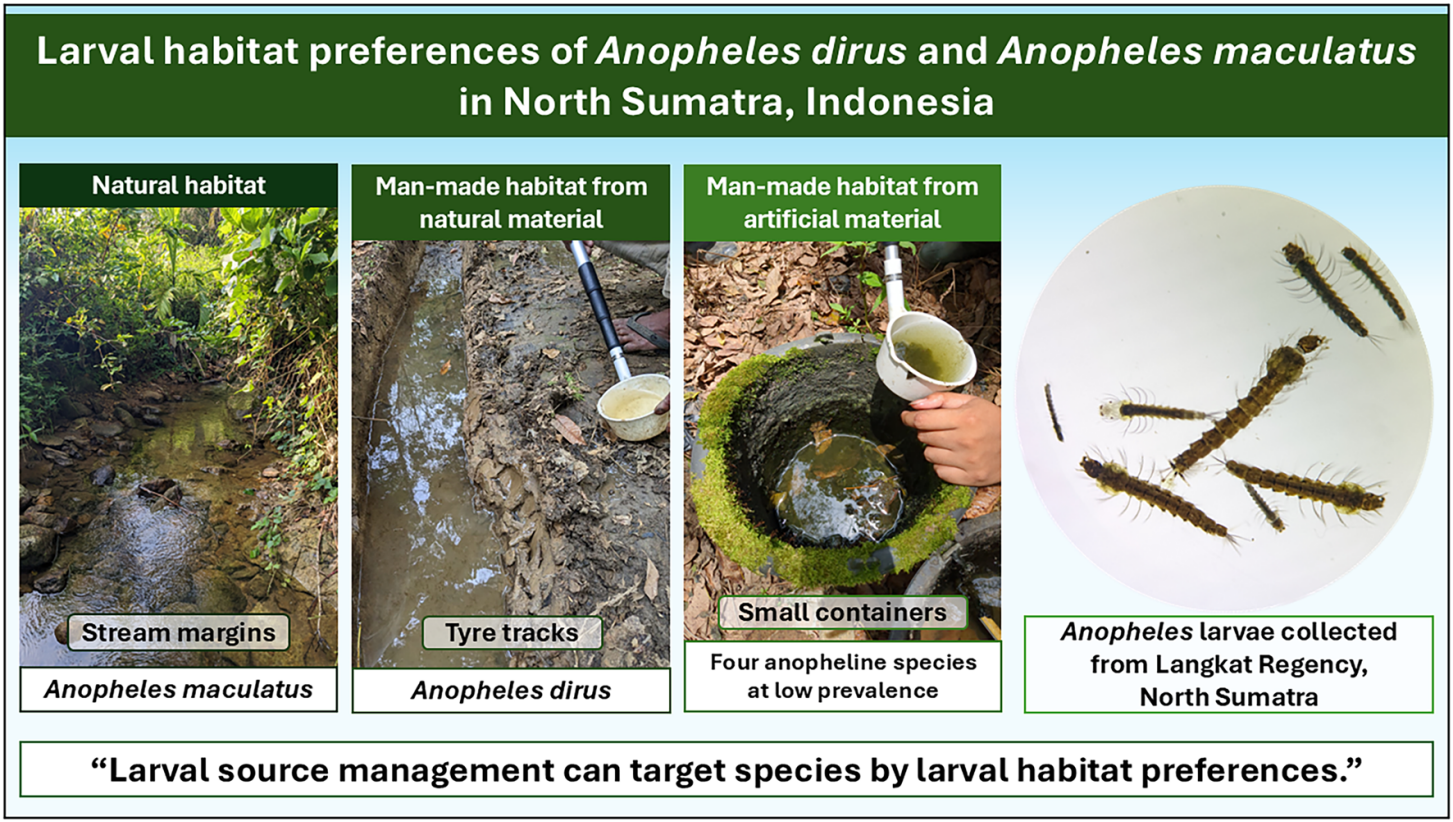

**Supplementary Information:**

The online version contains supplementary material available at 10.1186/s13071-026-07441-x.

## Background

Malaria transmission in Indonesia due to the major human malaria species *Plasmodium falciparum* and *Plasmodium vivax* has decreased and become increasingly localized, leading to a commitment by the Government of Indonesia to eliminate malaria by 2030 [1, 2]. However, human infections with *Plasmodium knowlesi*, a zoonotic malaria parasite that primarily infects nonhuman primates, particularly macaques, are increasingly reported. Over the past decade, *P. knowlesi* infections in humans have been molecularly confirmed across western Indonesia, including in the provinces of Aceh [3–5], North Sumatra [6, 7], Jambi [8], Central Kalimantan [9, 10], East Kalimantan [11], and South Kalimantan [12].

Mosquito species within the Leucosphyrus Group have been incriminated as the major vectors transmitting *P. knowlesi* to humans, despite only limited regional entomological data on the suspected vectors [13]. Several species within the Leucosphyrus Group are distributed in Indonesia, including *Anopheles leucosphyrus*, *Anopheles balabacensis*, *Anopheles dirus*, *Anopheles scanloni*, *Anopheles cracens*, and *Anopheles latens* [13–18]. However, molecular evidence of *P. knowlesi* (i.e., DNA detection) in Leucosphyrus mosquitoes in Indonesia has only been reported in a smaller subset of these species from limited testing to date, including *An*. *balabacensis* [16], *An*. *dirus* [18], and *Anopheles maculatus* [19]. In addition, *P. falciparum* and *P. vivax* DNA has been detected in *An. leucosphyrus* and *An. balabacensis* [14], making these species potential vectors of both zoonotic and human malaria.

The World Health Organization (WHO) has recommended malaria vector control strategies, insecticide-treated nets (ITNs), and indoor residual spraying (IRS), which has significantly reduced malaria transmission globally [1, 20, 21]. Insecticide-treated nets primarily target endophagic (indoor biting) vectors, and IRS primarily targets endophilic (indoor resting) vectors [20]. While ITNs and IRS can reduce transmission by exophagic and endophilic vectors, additional and complementary strategies are likely needed for effective control of these vectors [1, 22, 23]. As many malaria vector species in Southeast Asia, including both human and enzootic vectors, rarely bite or rest inside houses [24], understanding other behaviors of these malaria vectors is critical for selecting vector control strategies with the potential to be effective. Larval source management (LSM), recommended by the WHO as a supplemental malaria vector strategy, has the potential to control both indoor and outdoor biting and resting mosquitoes [25]. Larval source management is most effective where the larval habitats are few in number, fixed in location, and accessible, including where transmission is urban or seasonal [25].

There is limited information about the larval habitats of the human and zoonotic malaria vectors [13] in Indonesia and their vulnerability to LSM. The potential of LSM will be affected by human agricultural land use changes that can significantly impact the classes, subclasses, and abundance of mosquito larval habitats, directly influencing vector distributions and receptivity, and in so doing, affect the transmission potential of both human and zoonotic malaria [26–28]. Hence, this study aimed to define the distribution and abundance of different anopheline larval habitats to understand the potential of LSM in North Sumatra to control both human and zoonotic malaria vectors. The study was conducted in an area with both zoonotic and human malaria that had undergone significant land use changes over the past decade from deforestation, including the introduction and expansion of oil palm plantations [29, 30].

## Methods

### Study site

The study was undertaken in two of nine hamlets (dusuns) of Ujung Bandar Village in the Salapian Sub-district of Langkat Regency, North Sumatra Province, Indonesia. The study site was selected on the basis of two key criteria: (1) a predictive model incorporating environmental data on macaques, mosquito vectors, and land-use types to identify areas of high *P. knowlesi* transmission risk [29, 30] and (2) confirmed transmission of both zoonotic *P. knowlesi* and human malaria to humans through passive case detection (Lubis et al., unpublished data).

The main residential areas in both dusuns were surrounded by agricultural land, including oil palm and mixed plantations. In 2023, 38.3% of the Salapian Sub-district (22,173 ha) was agricultural land, comprised of oil palm (7846 ha), rubber (595 ha), coconut (31 ha), and cacao (11 ha) [31]. Ujung Bandar Village, with a population of over 1500 people, is 357 m above sea level and had an average annual rainfall of ~3400 mm in 2022 and 2023 [32, 33], with lower rainfall from January to June, mean annual outdoor temperature of 27.3 °C, 84.9% average humidity, and 47% sunlight exposure (Supplementary Material 1: Supplementary Fig. S1) [32, 33].

### Landscape classifications

The landscapes in which larval habitats were surveyed were categorized by visual inspection into four land-use types on the basis of the predominant human activity and vegetation present. These were: (1) residential areas, where most residents’ houses are closely located; (2) gardens, where food plants, including vegetables, are cultivated, usually near landowner houses; (3) oil palm plantations: monoculture cultivation, encompassing a heterogeneous mosaic of recently cleared, young, and mature oil palm trees; and (4) mixed plantations: agricultural areas where two or more plant species are cultivated. The area (m^2^) of each land-use type was calculated by recording GPS coordinates along the survey boundary, followed by creation and measurement of polygons in QGIS (version 3.34.14).

### Larval habitat surveys

After receiving verbal permission from landowners or village representatives, larval habitat surveys were conducted within a maximum 1-km radius from residential areas with the assistance of two local residents in July–August 2022 and March–April 2023 in both Dusun II (Pondok Cengkeh; 3.371720°N, 98.328547°E) and Dusun V (Deleng Payung; 3.341947°N, 98.335406°E). The geolocation (latitude and longitude in decimal degrees) of each habitat was recorded using a handheld GPS. A radius of up to 1 km was selected to focus on *Anopheles* larval habitats within the most epidemiologically relevant distance from human settlements, where human–vector contact is most likely to occur.

All water bodies encountered during the surveys were sampled for mosquito immature life stages. Sampling effort was standardized across habitats of varying size and sampling effort by dipping using 250 mL (125 mm diameter) standard dippers in which small habitats were sampled with up to ten dips. In larger habitats, sampling was performed at multiple sites (e.g., in fishponds, up to 25 dips were taken from each of the four sites, while in streams, sites were sampled at 5-m intervals with up to 10 dips taken per site along the stream margins). The number of mosquito immature stages and the total number of dips per habitat were recorded to enable calculation of the average number of immature mosquitoes per dip. Habitats with anopheline larvae present were defined as positive habitats, and without anopheline larvae as negative habitats. The GPS coordinates of all potential habitats (whether positive or negative) were recorded.

### Larval habitat classifications

Larval habitats were categorized into three primary classes: naturally occurring, man-made from natural materials, or man-made from artificial materials. Within each of these three classes, habitat subclasses categorized specific forms or examples of aquatic environments in which mosquito larvae were found.

Naturally occurring habitats were defined as water bodies formed without direct human or domestic animal input. Subclasses in this category included groundwater pools, rock holes, plant axils, and stream margins. Man-made habitats from natural materials were those created by human or domestic animal activity that altered the natural substrates. Subclasses included road or agricultural ditches, tire tracks, excavated fishponds, footprints of humans or livestock, and coconut shells modified after harvesting for copra or used to collect rubber sap. Man-made habitats from artificial materials were formed with synthetic or industrial materials, such as plastics, cement, rubber, metal, and glass. Subclasses in this category included discarded plastic containers, metal buckets, tires, and concrete-lined ditches. A full list of the habitat classes and subclasses, with detailed definitions and examples, is provided in Supplementary Material 2: Supplementary Table S1.

Each larval habitat was further characterized in the field by a range of physical (abiotic) and biological (biotic) features to understand habitat variation and its potential influence on mosquito presence. Abiotic parameters included:

- Substrate type: the material composition of the habitat floor (e.g., mud, rock, gravel, sand, concrete, metal, or plastic).
- Size: the approximate length of the water body, classified as < 0.5 m, 0.5–1 m, 1–2 m, 2–5 m, 5–10 m, and > 10 m.
- Water depth: very shallow (< 10 cm), shallow (10–30 cm), medium (30–60 cm), and deep (> 60 cm).
- Water flow: recorded as stagnant (no visible movement) or flowing (by observation of surface turbulence) where sampling occurred, often at habitat margins.
- Turbidity: classified as clear, cloudy, or opaque by allowing 50 mL of water placed in a clear tube to settle and be visually inspected.
- Water temperature, pH, and salinity, recorded at the time of collection, using a thermometer, pH meter, and refractometer, respectively.

Biotic parameters included:

- Canopy cover: the percentage of overhead vegetation shading the habitat, categorized as none, low (< 50% cover), or high (≥ 50% cover).
- Aquatic vegetation, including algae and emergent and submerged plants: the percentage composition of aquatic vegetation within the habitat (none, low < 50%, and high ≥ 50%).
- Aquatic fauna: the presence or absence of other invertebrates and vertebrates, including potential predators.

### Field laboratory processing of samples

Mosquito larvae and pupae samples were transported to a field laboratory in 50-mL labeled plastic containers. Larvae were identified to genus on the basis of standard morphological genus characteristics. *Anopheles* larval stages were classified and recorded as early (stages 1 and 2), late (stages 3 and 4), and a mixture of both early and late stages. Pupae and larvae were then preserved in 70% ethanol at room temperature.

### Identification of *Anopheles* larvae and mosquito pupae

Individual *Anopheles* larvae collected from each habitat were identified to species level using polymerase chain reaction (PCR) amplification of the internal transcribed spacer region II (ITS2) [34]. All mosquito pupae were similarly subjected to molecular assays for species identification. Representative PCR products from up to five mosquito immature samples with the same band size then underwent Sanger sequencing to confirm species identification using the Basic Local Alignment Search Tool (BLAST). Further validation for members of the Dirus Complex was performed using the Dirus Complex species identification PCR (DiCSIP) and Scanloni-specific PCR (SSP) assays [35], while multiplex PCR was used for species identification within the Maculatus Group [36]. Detailed procedures are provided in Supplementary Material 3: Supplementary Fig. S2.

Species-specific habitat associations were assigned on the basis of molecular identification of larvae and pupae collected from each habitat, with multiple species within the same habitat recorded as co-occurring.

### Statistical analysis

All field data were digitally recorded using the Ona platform (https://ona.io/) and the ODK application on an Android operating system. All data were exported to MS Excel (Microsoft Corp., Redmond, WA, USA) for statistical analyses with the R statistical environment (version 4.4.2), and all graphs were visualized by GraphPad (version 10.3.1). Generalized linear mixed models (GLMMs) compared the presence or absence of *Anopheles* larvae (binary model) and larval densities (negative binomial model) across different survey timepoints, dusun locations, land-use types, and habitat classes. To account for potential clustering of similar environmental characteristics within specific habitat classes, habitat subclass was included as a random effect in all GLMMs. Abiotic and biotic parameters of potential larval habitats, including perimeter, vegetation, canopy cover, water depth, turbidity, presence of predators, substrate type, water flow rate, temperature, and pH, were also analyzed in relation to the presence and density of *Anopheles* larvae. These analyses were performed using the *glmmTMB* package in R [37]. The random factor included variations in habitat subclasses observed during the survey. Distribution maps of *Anopheles* spp. larval habitats were created using QGIS (version 3.34.14).

## Results

### Distribution of *Anopheles* larval habitats and land use

**Fig. 1:**
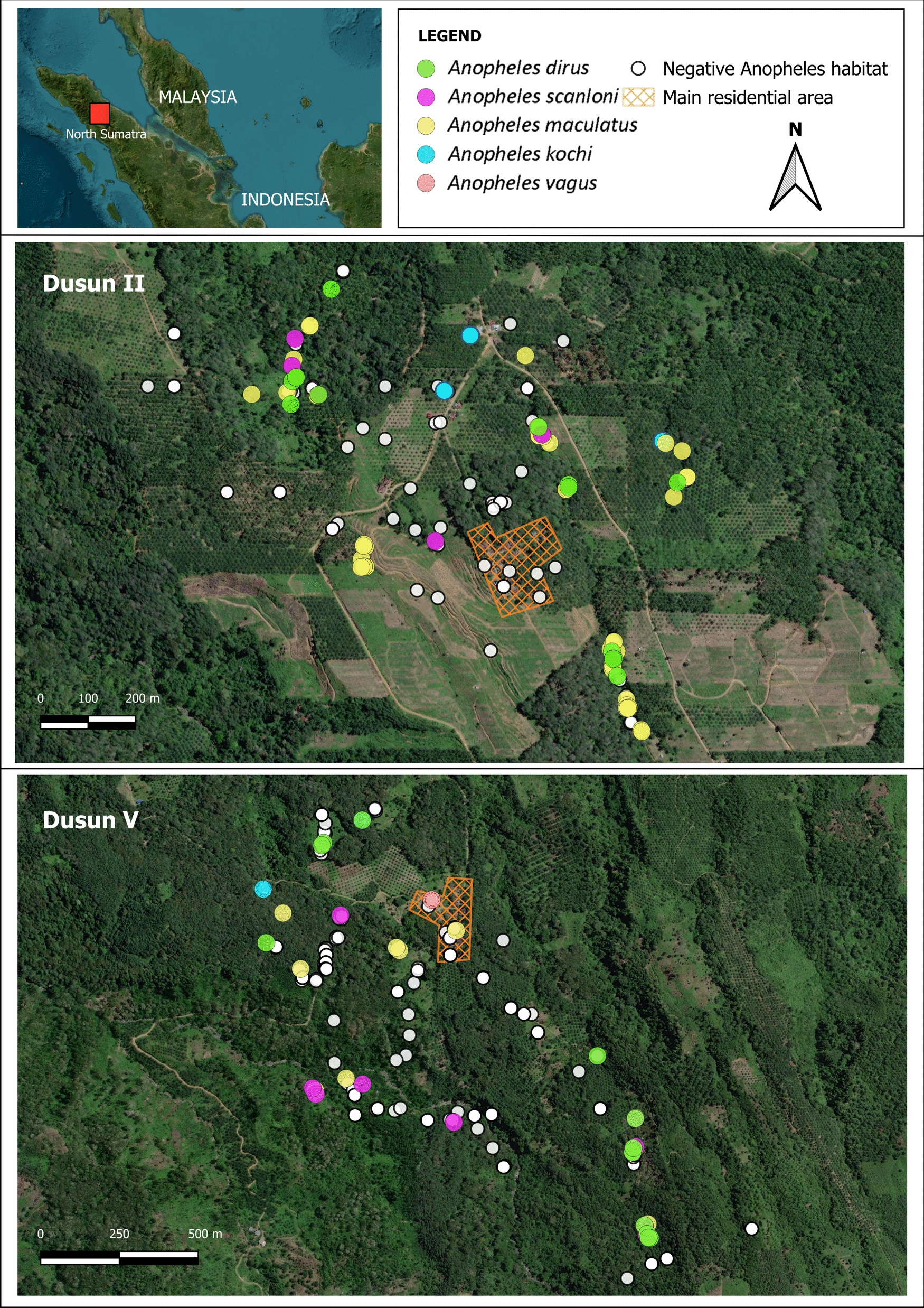
Spatial distribution of *Anopheles* larval habitats in Dusun II and V, Ujung Bandar Village, Salapian Sub-district, Langkat Regency, North Sumatra. The basemap was created with EsriWorld Imagery (WGS84) (source:https://www.arcgis.com/home/item.html?id=52bdc7ab7fb044d98add148764eaa30a)

A total of 338 aquatic habitats were found and characterized in a 1.348-km^2^ area during two surveys conducted in 2022 and 2023 across Dusuns II and V in Ujung Bandar Village, Salapian Sub-district, Langkat Regency, North Sumatra (Fig. 1; Supplementary Material 4: Supplementary Fig. S3). The largest land-use type surveyed was oil palm plantations, covering 0.805 km^2^ (59.7% of the surveyed area), followed by mixed plantations (0.477 km^2^, 35.4%), residential areas (0.06 km^2^, 4.5%), and gardens (0.006 km^2^, 0.4%) (Supplementary Material 5: Supplementary Fig S4). Most aquatic habitats (positive and negative) were found in mixed plantations (*n* = 195), followed by oil palm plantations (*n* = 107), main residential areas (*n* = 32), and gardens (*n* = 4). Some areas, particularly in the northeast of the main residential area in Dusun V, were not surveyed owing to limited accessibility or a lack of landowner permission (Supplementary Material 4: Supplementary Fig. S3). All surveyed larval habitats were freshwater (i.e., 0 ppm salt); thus, salinity was excluded from further analysis.

### Presence and density of *Anopheles* larvae across land-use type and habitat classes

**Fig. 2:**
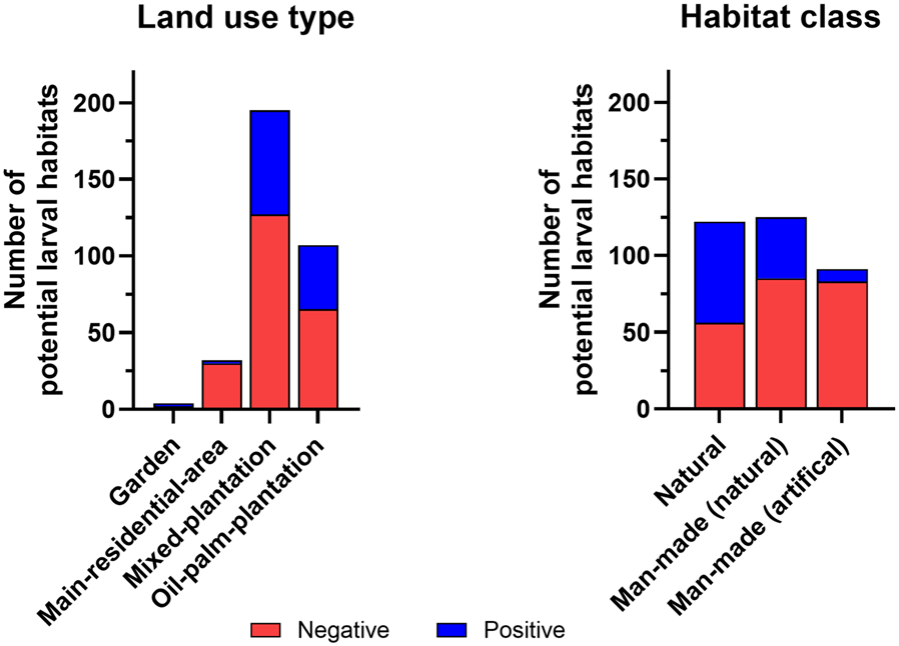
Number of potential larval habitats by land-use type and habitat class

A total of 1413 mosquito larvae were collected during the surveys with 291 (20.6%) larvae identified morphologically as anophelines, while 1122 (79.4%) were culicines. Overall, 114 (33.7%) potential larval habitats were positive for anopheline larvae. A total of 98 pupae were also collected. *Anopheles*-positive habitats were primarily located in mixed plantations (*n* = 68/195; 34.9%) and oil palm plantations (*n* = 42/107; 39.3%), with only two positive habitats each found in gardens (*n* = 2/4; 50%) and residential areas (*n* = 2/32; 6.25%) (Fig. 2). Although these two land-use types contained more positive habitats, this pattern likely reflects the greater area extent of mixed and oil palm plantations within the study area rather than a true ecological preference. Consistent with this, land-use type did not significantly influence either the presence (GLMM, *β* = 0.217, *P* = 0.433) or the density (GLMM, *β* = 0.329, *P* = 0.129) of *Anopheles* larvae (Table 1).

**Table 1:** Association of survey year, hamlets, land-use type, and habitat class with the presence and density of *Anopheles* larvae. Data were compared using GLMMs, with the habitat class as a random variable. The data were analyzed using two different distributions: a binary model (presence/absence) and a negative binomial model (density)

Parameters	Binary (presence/absence) model			Negative binomial (density) model		
	*β*	SE	*P*-value	*β*	SE	*P*-value
*Anopheles* genus						
(Intercept)	1.499	1.160	0.196	0.952	0.953	0.318
Year of survey	−0.345	0.292	0.236	−0.249	0.261	0.340
Hamlets	0.058	0.449	0.897	0.503	0.400	0.208
Land-use type	0.217	0.277	0.433	0.329	0.217	0.129
Habitat class	−1.450	0.531	0.006*	−1.421	0.409	0.001*
*Anopheles maculatus* larvae only						
(Intercept)	0.613	1.170	0.600	1.056	1.159	0.362
Year of survey	−0.054	0.371	0.884	−0.211	0.433	0.627
Hamlets	−0.386	0.570	0.498	0.913	0.648	0.159
Land-use type	0.333	0.331	0.313	0.428	0.342	0.211
Habitat class	−1.452	0.508	0.004*	−2.168	0.449	< 0.001*
*Anopheles dirus* larvae only						
(Intercept)	−0.982	1.144	0.391	−0.628	1.172	0.592
Year of survey	−0.089	0.404	0.827	0.106	0.433	0.806
Hamlets	0.379	0.614	0.538	2.180	0.760	0.004*
Land-use type	−0.070	0.408	0.864	0.314	0.438	0.473
Habitat class	−1.084	0.517	0.036*	−2.027	0.625	0.001*

^*^Statistically significant. SE, standard error

Habitat class, in contrast, had a strong association with both presence (GLMM, *β* = −1.450, *P* = 0.006) and density (GLMM, β = −1.421, *P* = 0.001) of *Anopheles* larvae. Larval occurrence was highest in naturally occurring habitats and man-made habitats created from natural materials, whereas artificial man-made habitats contributed relatively few anopheline detections (Fig. 2; Table 1). A detailed breakdown of larval density and positivity rates across specific habitat subclasses is presented in Table 2. Notably, stream margins (77.4% positive, 0.74 larvae/dip) and rock holes (60% positive, 0.81 larvae/dip) showed the highest larval positivity and densities, while artificial man-made habitats, including small containers, were infrequently positive and had low larval densities.

**Table 2:** Larval habitats of anophelines in Dusun II and V in Ujung Bandar Village, Salapian Sub-district, Langkat Regency, North Sumatra, Indonesia

Habitat class	Habitat subclass		Positive *Anopheles* habitats		Larval density per dip
	Containers (no.)		(Positive)	(%)	(Mean ± SE)
Natural	Groundwater pools	38	9	23.7	0.46 ± 0.05
	Rock holes	15	9	60.0	0.81 ± 0.05
	Stream margins	62	48	77.4	0.74 ± 0.1
	Tree holes or plant axils	7	0	0.0	NA
Man-made from natural materials	Coconut shells	9	1	11.1	0.05 ± 0.00
	Ditches	8	5	62.5	0.31 ± 0.06
	Fishponds	16	4	25.0	0.07 ± 0.01
	Footprints and hoof prints	1	0	0.0	NA
	Dug holes	22	3	13.6	1.18 ± 0.03
	Tire tracks	69	27	39.1	0.32 ± 0.02
Man-made from artificial materials	Drain	3	0	0.0	NA
	Large containers (> 25 L) (plastic and metal drums, plastic and concrete water containers)	16	0	0.0	NA
	Small containers (< 10 L) (buckets, jerrycans, plastic sheets, plastic food containers)	65	8	12.3	0.85 ± 0.04
	Tires	7	0	0.0	NA

NA, not applicable

Larval presence was positively associated with aquatic vegetation (GLMM, *β* = 0.583, *P* = 0.020); specific substrate types such as mud, rock, or gravel (GLMM, *β* = 0.354, *P* = 0.024); flowing water at sampling points (GLMM, *β* = 1.800, *P* < 0.001); and alkaline pH (range 5.2–8.6, GLMM, *β* = 1.169, *P* < 0.001) (Table 3). In contrast, deeper water bodies were negatively associated with larval presence (GLMM, *β* = −0.422, *P* = 0.034) (Table 3). Larval density for *Anopheles* species followed a similar pattern. Higher larval densities were associated with aquatic vegetation (GLMM, *β* = 0.464, *P* = 0.024), muddy or organic substrates (GLMM, *β* = 0.283, *P* = 0.025), flowing water (GLMM, *β* = 0.794, *P* = 0.019), and higher pH (GLMM, *β* = 0.988, *P* < 0.001). In contrast, turbid water (GLMM, *β* = -0.672, *P* = 0.036) and warmer water temperatures (GLMM, *β* = −0.152, *P* = 0.036) were negatively associated with larval density (Table 3).

**Table 3:** Association of abiotic and biotic parameters with the presence and density of *Anopheles* larvae. Data were compared using GLMMs, with the habitat class as a random variable. The data were analyzed using two different distributions: a binary model (presence/absence) and a negative binomial model (density)

Parameters	Binary (presence/absence) model			Negative binomial (density) model		
	*β*	SE	*P*-value	*β*	SE	*P*-value
All *Anopheles* larvae						
(Intercept)	−10.232	3.238	0.002*	−6.080	2.640	0.021*
Perimeter	0.140	0.171	0.414	0.093	0.130	0.476
Vegetation	0.583	0.250	0.020*	0.464	0.205	0.024*
Canopy cover	0.266	0.183	0.146	0.196	0.156	0.208
Water depth	−0.422	0.199	0.034*	−0.054	0.132	0.680
Turbidity	−0.615	0.364	0.091	−0.672	0.320	0.036*
Predators	−0.350	0.340	0.303	−0.334	0.239	0.163
Substrate	0.354	0.156	0.024*	0.283	0.126	0.025*
Flow rate	1.800	0.474	< 0.001*	0.794	0.339	0.019*
Temperature	-0.101	0.095	0.286	−0.152	0.072	0.036*
pH	1.169	0.315	< 0.001*	0.988	0.264	< 0.001*
*Anopheles maculatus* larvae only						
(Intercept)	−8.671	3.878	0.025*	−5.697	4.124	0.167
Perimeter	0.046	0.196	0.815	0.049	0.209	0.815
Vegetation	0.770	0.279	0.006*	0.780	0.336	0.021*
Canopy cover	0.258	0.228	0.256	0.490	0.247	0.048*
Water depth	−0.123	0.207	0.551	0.209	0.216	0.333
Turbidity	−0.171	0.454	0.706	−0.958	0.531	0.071
Predators	0.202	0.418	0.628	0.143	0.396	0.717
Substrate	0.193	0.155	0.212	0.271	0.187	0.146
Flow rate	1.421	0.458	0.002*	0.864	0.547	0.114
Temperature	−0.056	0.119	0.636	−0.267	0.113	0.019*
pH	0.511	0.392	0.193	0.954	0.437	0.029*
*Anopheles dirus* larvae only						
(Intercept)	−7.392	4.835	0.126	−6.761	5.238	0.197
Perimeter	0.365	0.230	0.113	0.070	0.210	0.739
Vegetation	−0.083	0.359	0.817	−0.258	0.389	0.508
Canopy cover	0.067	0.259	0.794	−0.372	0.230	0.106
Water depth	−0.930	0.395	0.019*	−0.704	0.347	0.042*
Turbidity	0.030	0.518	0.954	0.302	0.548	0.581
Predators	−0.447	0.467	0.339	−0.932	0.354	0.008*
Substrate	0.141	0.202	0.484	0.139	0.234	0.551
Flow rate	0.237	0.538	0.659	0.312	0.594	0.599
Temperature	−0.153	0.142	0.282	−0.066	0.159	0.677
pH	1.253	0.506	0.013*	1.301	0.529	0.014*

^*^Statistically significant

### *Anopheles* larvae composition and habitat distribution

A total of 242 *Anopheles* larvae were identified to species using molecular techniques (Supplementary Material 6: Supplementary Fig. S5). The predominant species was *Anopheles maculatus* (65.3%, *n* = 158), followed by *Anopheles dirus* (21.9%, *n* = 53), *Anopheles scanloni* (7%, *n* = 17), *Anopheles kochi* (5.4%, *n* = 13), and *Anopheles vagus* (0.4%, *n* = 1). Of the 50 pupae identified molecularly, 3 were *An. maculatus* (6.0%), while the remaining were culicine species: *Aedes albopictus* (28.0%, *n* = 14), *Armigeres subalbatus* (12.0%, *n* = 6), and *Culex* spp. (54.0%, *n* = 27).

**Fig. 3:**
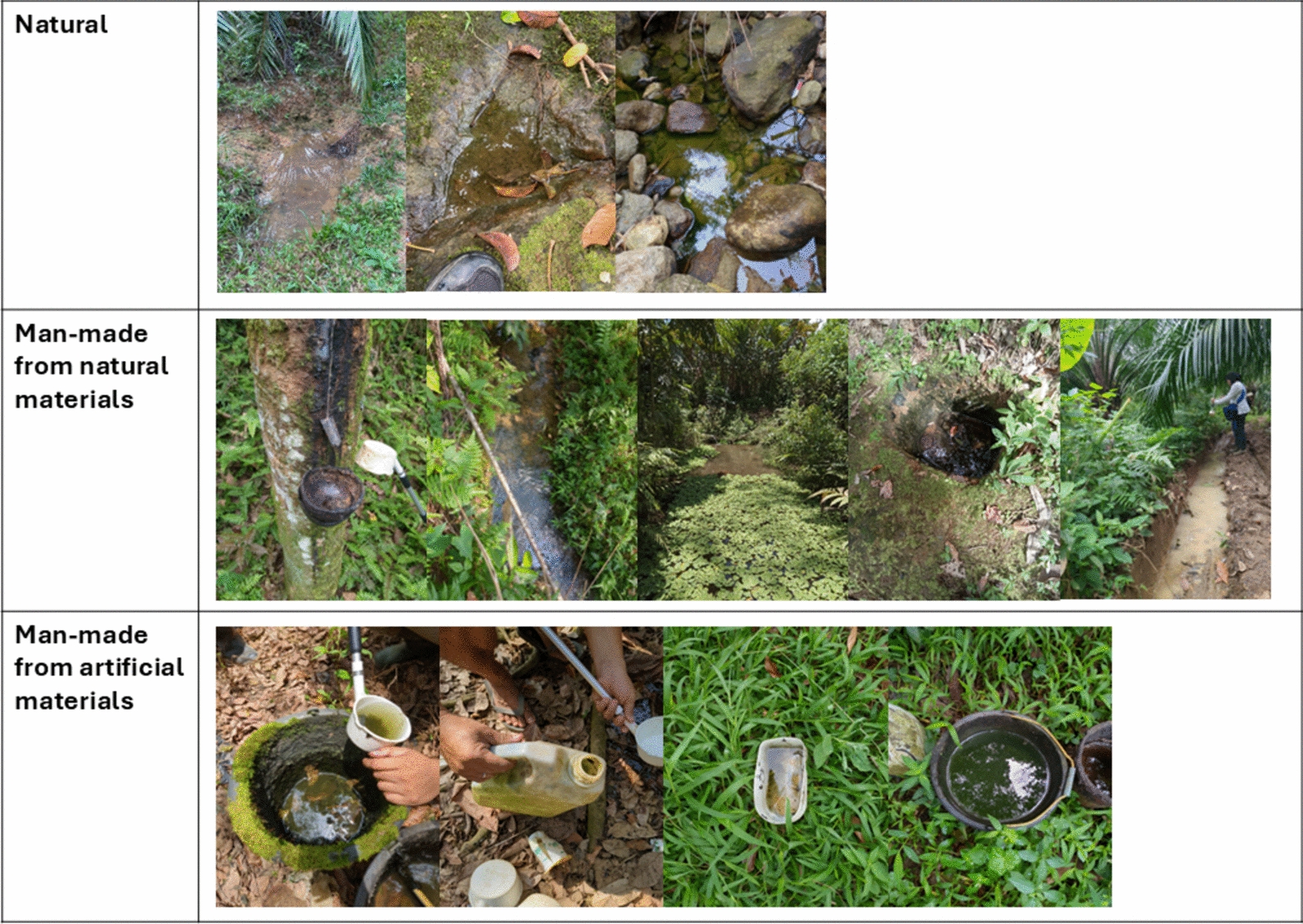
Larval habitats with positive *Anopheles* larvae. Natural habitats (left to right: ground pools, rock holes, and stream margins); man-made from natural materials (left to right: coconut shells, ditch, fishpond, dug holes, and tire tracks); man-made from artificial materials (small plastic containers < 10 L)

*Anopheles maculatus* (*n* = 64), *An*. *dirus* (*n* = 33), *An. scanloni* (*n* = 13), and *An. kochi* (*n* = 7) were detected across a variety of larval habitats, spanning natural habitats, man-made habitats from natural materials, and man-made habitats from artificial materials (Table 4; Fig. 3). Natural stream margins were the most frequently occupied habitat type for *An. maculatus* (*n* = 39: 61%), with *An. dirus* most commonly found in tire tracks (*n* = 14; 42%). *Anopheles scanloni* was most frequently detected in stream margins (*n* = 4; 31%), whereas *An. kochi* and *An. vagus* were observed at low frequencies across a limited range of habitats (Table 4; Fig. 3).

**Table 4:** *Anopheles* species larval habitats in Dusun II and V, Ujung Bandar Village, Salapian Sub-district, Langkat Regency, North Sumatra, Indonesia

Habitat class	Habitat subclass	No. of occurrences*	Number of larval habitats occupied				
			*An. maculatus*	*An. dirus*	*An. scanloni*	*An.* *kochi*	*An.* *vagus*
Natural	Groundwater pools	38	3	4	2	0	0
	Rock holes	15	5	4	0	1	0
	Stream margins	62	39	7	4	0	0
	Total	115	47	15	6	1	0
Man-made from natural materials	Coconut shells	9	1	0	0	0	0
	Ditches	8	3	2	0	0	0
	Fishponds	16	3	0	1	0	0
	Dug holes	22	2	1	0	1	0
	Tire tracks	69	7	14	3	2	1
	Total	124	16	17	4	3	1
Man-made from artificial materials	Small containers (< 10 L)	65	1	1	3	3	0
Total		65	1	1	3	3	0
Overall total		304	64	33	13	7	1

^*^No. of occurrences refers to the total number of mosquito larval habitats recorded during the survey, regardless of whether they were occupied by *Anopheles* larvae

Multiple species were detected as co-occurring within the same habitat class: *An. dirus*, *An. scanloni*, and *An. kochi* in two tire tracks; *An. dirus*, *An. scanloni*, and *An. maculatus* in two stream margin sites; and *An*. *dirus* and *An. kochi* in both a natural rock hole and a human-dug hole. Four *Anopheles* species (*An. maculatus*, *An. dirus*, *An. scanloni*, and *An. kochi*) were observed in artificial containers (e.g., plastic buckets and jerrycans [< 10L]) in both mixed and oil palm plantations (Table 4). Tire tracks were the only habitat type in which all five *Anopheles* species were detected (Table 4).

### The presence and density of *An. maculatus *and *An. dirus* larvae

The two most common *Anopheles* species were modeled separately to evaluate presence and density controlling for land-use type, habitat class and subclass (Table 1), and biotic/abiotic parameters (Table 3), respectively.

The presence of both *Anopheles* larvae differed among habitat classes, with man-made artificial habitats showing significantly lower odds of larval presence relative to the reference habitat classes (*An. maculatus* GLMM, *β* = −1.452, *P* = 0.004; *An. dirus* GLMM, *β* = −1.084, *P* = 0.036; Table 1). For *An. maculatus*, positive associations were observed with increasing vegetation (GLMM, *β* = 0.770, *P* = 0.006) and the presence of flowing-water habitats (GLMM, *β* = 1.421, *P* = 0.002), with larvae occurring primarily in stream margin habitats with aquatic vegetation (Table 3). In contrast, *An. dirus* larvae were more likely to occur in shallow water (GLMM, *β* = −0.930, *P* = 0.019) and under more alkaline pH conditions (GLMM, *β* = 1.253, *P* = 0.013) (Table 3; Supplementary Material 7 and 8: Supplementary Figs. S6 and S7).

Larval densities of both *An. maculatus* and *An. dirus* differed significantly among habitat classes, with artificial man-made habitats supporting significantly lower larval densities compared with naturally occurring habitats and man-made habitats from natural materials (Table 1). In addition, *An. dirus* larval density showed a significant association with dusuns (GLMM, *β* = 2.180, *P* = 0.004), with higher densities observed in Dusun V than in Dusun II (Table 1). These associations indicate spatial variation in larval densities across the study area but do not imply causality, as habitat class and habitat subclass were included in the models to account for underlying environmental heterogeneity.

*Anopheles maculatus* larval density was positively associated with aquatic vegetation (GLMM, *β* = 0.780, *P* = 0.021), canopy cover (GLMM, *β* = 0.490, *P* = 0.048), and pH (GLMM, *β* = 0.954, *P* = 0.029), and negatively associated with temperature (GLMM, *β* = −0.267, *P* = 0.019) (Table 3).

*Anopheles dirus* larvae showed increased density in shallow water (GLMM, *β* = −0.704, *P* = 0.042), at higher pH (GLMM, *β* = 1.301, *P* = 0.014), and in the absence of aquatic predators (GLMM, *β* = −0.932, *P* = 0.008) (Table 3). Observed aquatic predators included tadpoles, dragonfly nymphs, water striders, and *Toxorhynchites* larvae and fish.

### Spatial distributions of larval habitats by *An. maculatus* and *An. dirus*

**Fig. 4:**
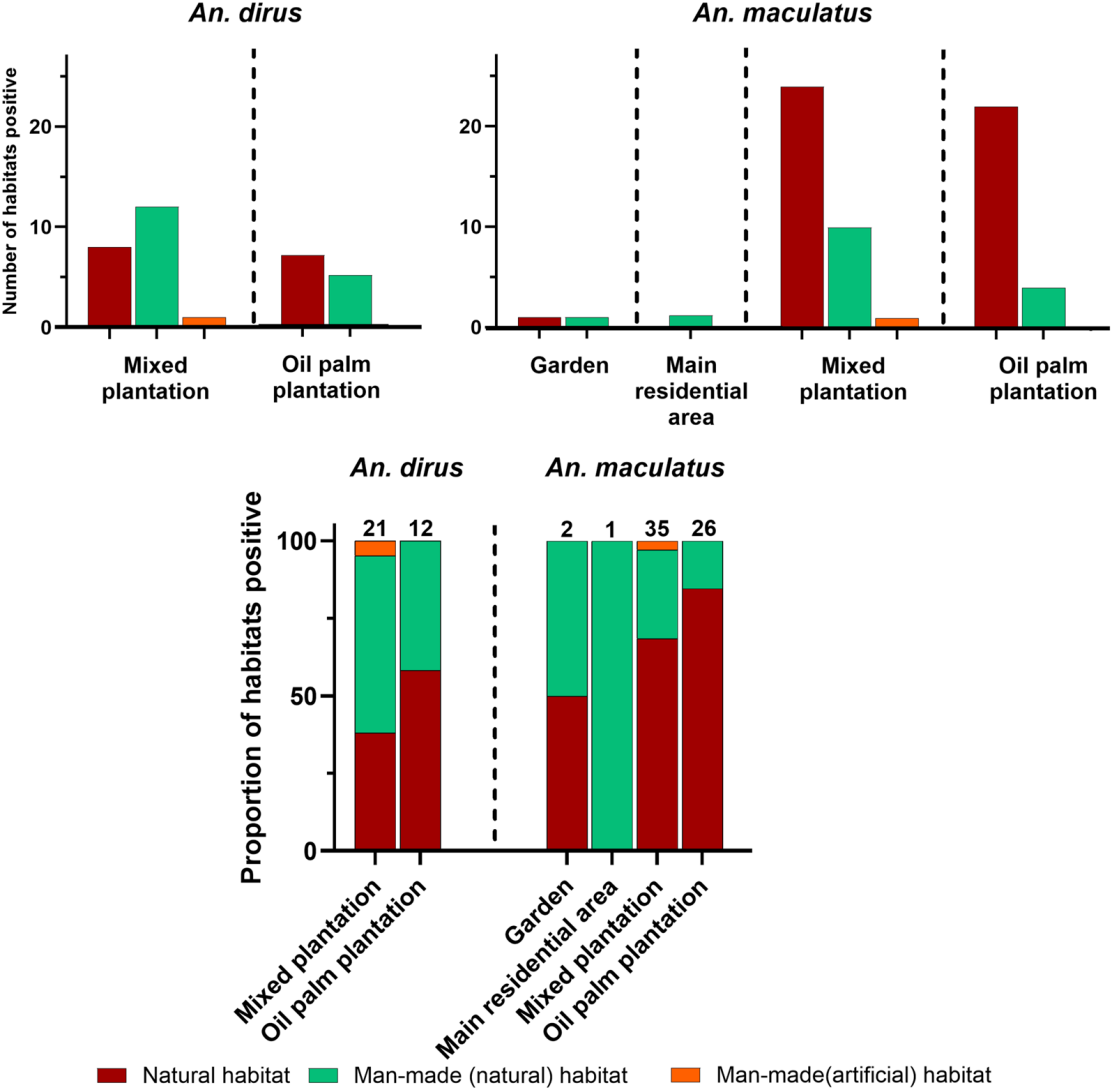
The number of positive larval habitats by species in land-use type and habitat class

Land-use type was not independently associated with the presence of *Anopheles* larvae in multivariable models, for *Anopheles maculatus* and *An. dirus* (Table 1); spatial patterns for these two species are described here when adjusted for surveyed area (Table 5). Garden habitats, although limited in size, had the highest larval habitat density for *An. maculatus* (333 positive habitats/km^2^), followed by mixed plantations (73 habitats/km^2^), oil palm plantations (32 habitats/km^2^), and residential areas (17 habitats/km^2^) (Table 5; Supplementary Material 4 and 5: Supplementary Figs. S3 and S4). In absolute numbers, *An. maculatus* larvae were found most frequently in mixed plantations (*n* = 35), followed by oil palm plantations (*n* = 26), gardens (*n* = 2), and residential areas (*n* = 1), reflecting the larger land area of mixed plantations and oil palm plantations (Fig. 4). *Anopheles dirus* larvae were not found in residential areas or gardens (Fig. 4) but were most commonly found in mixed plantations (44 positive habitats/km^2^), followed by oil palm plantations (15 habitats/km^2^) (Table 5).

**Table 5:** Density of *An. maculatus* and *An. dirus* larval habitats across different land-use types

Land-use type	Surveyed area (km^2^)	*An. maculatus* larval habitat		*An. dirus* larval habitat	
		Number (*n*)	Number (*n*)	Number (*n*)	Density (*n*/km^2^)
Gardens	0.006	2	333	0	0
Residential areas	0.06	1	17	0	0
Mixed plantations	0.477	35	73	21	44
Oil palm plantations	0.805	26	32	12	15

## Discussion

This study comprehensively assessed *Anopheles* larval habitats in Ujung Bandar Village, North Sumatra, integrating land use, habitat class, and habitat subclass to identify key determinants of larval presence and density. While multiple land-use types were surveyed, larval occurrence and density were most strongly associated with habitat class and subclass rather than broader land-use categories, highlighting the importance of micro-environmental conditions in shaping larval habitat suitability. Naturally occurring habitats and man-made habitats created from natural materials supported the majority of *Anopheles* larvae, whereas artificial man-made habitats contributed minimally. These findings underscore the relevance of habitat-focused larval source management (LSM), where targeting specific habitat types and micro-environments may be more effective than land-use-based interventions.

The LSM remains an important component of many integrated vector control strategies, particularly in settings where larval habitats are few, fixed, and identifiable [25, 38]. Targeting immature stages of mosquitoes can reduce adult vector populations and complement existing interventions such as insecticide-treated nets and indoor residual spraying [25, 39]. However, the effectiveness of LSM is highly dependent on local ecological conditions, including habitat accessibility, stability, and productivity, as well as operational feasibility [25, 40].

Five *Anopheles* larval species were identified: *An. maculatus*, *An. dirus*, *An. scanloni*, *An. kochi*, and *An. vagus.* These results were consistent with adult mosquito species caught by human landing catch (HLC) from a concurrent longitudinal study in the same locations [41]. Three of these species were previously recognized as human malaria vectors in Indonesia [42]: *An. kochi* is a secondary vector in Central Java, northern Sumatra, and Sulawesi [42]; *An. vagus* is a secondary vector in Banten and East Nusa Tenggara (Sumba) [43, 44]; and *An. maculatus *var. *manoreh* are primary vectors in Central Java (Menoreh Hills) [45] and secondary vectors in western Indonesia, including Yogyakarta and northern and southern Sumatra [42]. *Anopheles maculatus* s.s. is a potential vector for zoonotic malaria with DNA detected (i.e., *Plasmodium inui* and *P. knowlesi*) from separated thorax samples in North Sumatra (Langkat) [19]. *Anopheles dirus* and *An. scanloni* are members of the Dirus Complex within the Leucosphyrus Group, with both species recently molecularly confirmed for the first time in Indonesia [18]. *Plasmodium* species DNA detected in *An. dirus* further supports this species as a probable vector of zoonotic, enzootic, and human malaria in Langkat, North Sumatra [18]. While the malaria vector status of *An. scanloni* in Indonesia remains unknown, this species is a secondary vector in Thailand [46, 47].

Spatial patterns in larval habitat use varied markedly by *Anopheles* species, reflecting species-specific ecological differences and adaptability to environmental conditions. *Anopheles maculatus*, the most frequently detected species, exhibited a strong preference for natural aquatic environments, especially stream margins, with these habitats characterized by slow-flowing water, moderate tree canopy cover, muddy or rock substrates, and aquatic vegetation—features positively associated with larval presence and density in the multivariable model. These ecological preferences are consistent with historical reports of *An. maculatus* s.l. breeding in clean, partially shaded running water in foothill or forested environments across Peninsular Malaysia and western Indonesia [46]. In contrast, *An. dirus* was more ecologically adaptable, frequently using habitats formed from natural substrates by human activities, such as tire tracks as well as natural habitats. These shallow, sun-exposed depressions, common in mixed and oil palm plantations, often collect transient rainwater and were associated with environmental parameters such as alkaline pH and absence of aquatic predators, also found to be associated with larval presence and density. While both species were rarely found in artificial habitats (e.g., plastic buckets, jerrycans), their occasional occurrence, even in low numbers, particularly for *An. dirus*, suggests some emerging behavioral capacity to exploit novel environments. These findings align with previous research in Southeast Asia [42, 48], but provide new evidence for habitat use in a region where *P. knowlesi* zoonotic transmission has been detected [6].

Although land-use type, such as oil palm, mixed plantations, or gardens, did not emerge as an independent predictor of *Anopheles* larvae presence or density in multivariable models, habitat class, particularly the distinction between natural and artificial environments, proved to be a robust ecological determinant, consistent with previous studies in Southeast Asia demonstrating that larval occurrence is more strongly structured by local habitat characteristics than by broad land-use categories alone [26, 48–50]. Natural habitats, including stream margins and man-made habitats from natural materials, such as tire tracks and rock holes, supported higher densities of both *An. maculatus* and *An. dirus*. Ecological conditions such as shaded vegetation, stagnant or flowing water, and suitable substrates favored the presence and abundance of anophelines. These abiotic and biotic parameters emerged as significant predictors in the multivariable models, underlining the complex interplay between vector ecology and habitat characteristics. The absence of consistent patterns for *Anopheles* abundance across individual land-use categories may be explained by small sample sizes in less prevalent land-use types (e.g., gardens, residential areas) and overlapping vegetation structures across study sites or mixed-use areas, which may mask broader land-use effects. However, a study from Sabah, Malaysia, showed that certain land-use types, such as eucalyptus plantations and oil palm plantations, can support significantly higher mosquito numbers than restored native dipterocarp forest [51, 52], likely reflecting differences in vegetation structure, shading, and microhabitat availability rather than land-use category alone. In line with our findings, this highlights the need for fine-scale investigations of habitat conditions influencing *Anopheles* breeding in North Sumatra.

The detection of *An. dirus* and *An. scanloni* primarily outside residential areas is consistent with their known ecological associations with forested and peri-domestic environments [48]. However, given that the flight range of *Anopheles* mosquitoes averages around 500 m [53] but can extend up to 1.5 km for *An. dirus* s.l. [54, 55], adults emerging from larval habitats up to 1–2 km from households may still pose a significant risk of human–vector contact [26, 27], particularly in areas with overlapping human and macaque activity [56]. However, in a parallel HLC study, there was an absence of significant biting by *An. dirus* and *An. maculatus* in residential areas [41], suggesting spatial or ecological barriers to host-seeking behavior in those settings. One plausible contributing factor is the lower presence of wild macaques, the suspected natural blood meal sources of *An. dirus*, within the main residential area of the two dusun areas surveyed. Studies have shown that macaques are primarily active in rubber and oil palm plantations and that members of the Leucosphyrus Group tend to maintain their presence near macaques [26, 57].

A key operational insight from this study is the contrasting feasibility of LSM as a vector control tool for different *Anopheles* species, based on their larval habitat preferences. LSM is generally considered most effective in areas where larval habitats are limited in number, fixed in location, and easily accessible, particularly in settings with seasonal transmission or periurban environments [25, 38]. In this context, *An. dirus* may represent a more suitable target for intervention: The species frequently utilized man-made habitats from natural materials such as tire tracks, which are often clustered, stable, and amenable to physical modification, drainage, or larviciding, consistent with a prior study that also commonly found *An. dirus* s.l. in ground pools, including in tire tracks [48]. These characteristics make LSM a potentially feasible and cost-effective strategy for reducing *An. dirus* populations in Langkat, North Sumatra.

In contrast, LSM appears considerably more challenging for controlling *An. maculatus*, whose larvae were primarily found in natural stream margins, habitats that are flowing, spatially dispersed, and difficult to treat consistently. The ecological characteristics of these sites, including their complexity, scale, and remoteness, pose significant logistical and financial barriers to practical application of LSM tools. Similar findings were reported at various sites in Indonesia [42] and Malaysia [50], which found that *An. maculatus* s.l. larvae were predominantly in natural habitats such as stream margins, as was found here.

Environmental management strategies targeting mosquito larval sites in hilly areas, such as sub-soil drainage, covered channels, and stream-flushing systems, were documented in various regions [58], including historically in West Malaysia since the early 1900s [59]. These interventions effectively reduced larval habitat sites of *An. maculatus* and other vector species in specific hill regions where terrain, hydrology, and infrastructure facilitated sustained implementation [58, 59]. However, the range of ecologies in the present study area differs considerably. Here, *An. maculatus* larval habitat sites are widely scattered along natural stream margins without existing drainage infrastructure, in locations that are difficult to access and subject to seasonal variability. Implementing large-scale engineering solutions similar to those used in other hill regions would present significant logistical, financial, and operational challenges. Therefore, despite historical successes elsewhere, the feasibility of LSM-based large-scale environmental control for *An*. *maculatus* in North Sumatra seems limited.

The detection of four *Anopheles* species, *An. maculatus*, *An. dirus*, *An. scanloni*, and *An. kochi*, in artificial containers, although rare, is a noteworthy finding that suggests potential behavioral plasticity and possible adaptation to anthropogenic environments. Such trends warrant close surveillance, as they could signal a shift in vector ecology that expands larval habitat sites into urban or periurban zones. The presence of *Anopheles* larvae in artificial habitats is most notably seen in the urban malaria vector, *Anopheles stephensi,* that oviposits in a diverse array of artificial sites in urban environments [60]. Similarly, *An*. *vagus* larvae were reported in tires, drums, and dinghies in Indonesia [42]. However, given the low frequency of anopheline larvae in artificial containers in the present study, surveillance and control efforts are likely to be more effectively prioritized toward man-made habitats from natural materials, which contributed more substantially to overall larval occurrence and density.

The density and distribution of *An. dirus* and *An. maculatus* larval habitats reflect both ecological availability and the detection sensitivity of larval surveillance. Detection rates of mosquito larvae are influenced by sampling effort, including the number of dips per site, number of collectors, site accessibility, and whether locations are fixed or opportunistic [61]. While greater effort improves detection sensitivity, it may limit geographic coverage owing to time and staffing constraints. This trade-off is particularly relevant in extensive oil palm and mixed plantations, which in this study supported the highest numbers of *An. dirus* and *An. maculatus* habitats. Operational assessments are therefore essential to balance thoroughness and coverage, guiding resource allocation and goal setting [21].

These considerations reinforce the importance of detailed larval habitat mapping to guide targeted vector control. Where appropriate, LSM for malaria control should consider prioritizing man-made habitats from natural materials that are accessible and relatively stable. More broadly, the results support integrated vector management strategies that combine entomological surveillance, habitat modification, and responsive control measures tailored to species-specific behaviors and environmental contexts. However, this study had several limitations. Larval surveys were restricted to selected land-use types, and sampling effort was not evenly distributed across land-use types or habitat classes owing to access constraints. Although larval density was expressed per dip to improve comparability across habitats, variation in sampling intensity among habitats may still influence detection probability and density estimates. These factors should be considered when interpreting habitat-specific associations. In addition, oil palm plantations were treated as a single land-use category despite heterogeneity in planting stages that may influence aquatic habitat availability. These limitations warrant cautious interpretation and highlight the value of complementary longitudinal or spatially stratified studies.

## Conclusions

This study highlights the importance of habitat class in shaping the distribution and density of *Anopheles* larvae in North Sumatra, particularly for *An. dirus* and *An. maculatus*. Naturally occurring and man-made habitats from natural materials consistently supported larval populations. The occasional detection of *An. maculatus*, *An. dirus*, *An. scanloni*, and *An. kochi* in artificial containers, suggests limited but notable adaptation to human-associated environments. These findings have practical implications for vector control: *An. dirus*, which frequently used accessible habitats such as tire tracks, may present a potentially suitable target for LSM. However, occurrence alone does not necessarily reflect larval productivity or the relative contribution of specific habitats to adult mosquito populations, and this should be considered when interpreting the feasibility of LSM. In contrast, *An. maculatus* was primarily found in less accessible stream margins. Continued larval surveillance is essential to monitor these patterns, particularly in landscapes undergoing rapid environmental change, and to guide locally adapted vector control strategies informed by species-specific ecological preferences.

## Supplementary Information

Supplementary Material 1.

Supplementary Material 2.

Supplementary Material 3.

Supplementary Material 4.

Supplementary Material 5.

Supplementary Material 6.

Supplementary Material 7.

Supplementary Material 8.

## Data Availability

The datasets supporting the conclusions of this article are available at the Research Data JCU repository: https:/doi.org/10.25903/9vfm-rb40.

## Abbreviations

*An.*: *Anopheles*
BLAST: Basic Local Alignment Search Tool
DiCSIP: Dirus complex species identification PCR
GLMMs: Generalized linear mixed models
HLC: Human landing catch
LSM: Larval source management
*P.*: *Plasmodium*
PCR: Polymerase chain reaction
s.l.: Sensu lato
s.s.: Sensu stricto
SSP: Scanloni-specific PCR
