# Supplementary material for: Larval habitat preferences of Anopheles dirus and Anopheles maculatus in North Sumatra, Indonesia
Source: Parasit Vectors. 2026 May 18;19:286. doi: 10.1186/s13071-026-07441-x (PMC13348638; doi:10.1186/s13071-026-07441-x)
Supplement: Supplementary file 1 — Supplementary Material 1. [file 13071_2026_7441_MOESM1_ESM.docx]

**Additional file 1**


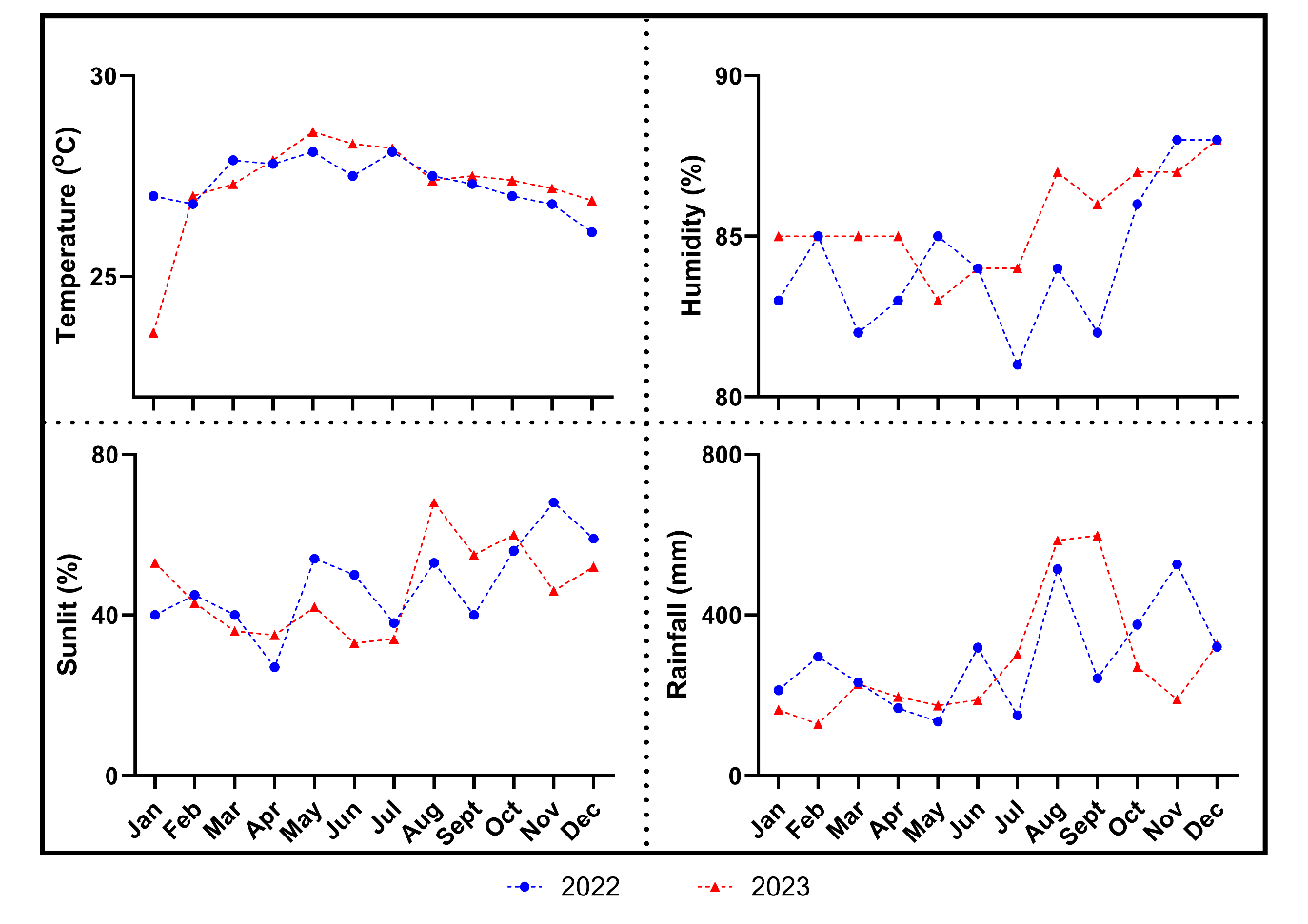


**Fig. S1 Environmental conditions of Ujung Bandar Village in 2022 and 2023** (temperature, humidity, sunlight and rainfall).
