# Supplementary material for: Larval habitat preferences of Anopheles dirus and Anopheles maculatus in North Sumatra, Indonesia
Source: Parasit Vectors. 2026 May 18;19:286. doi: 10.1186/s13071-026-07441-x (PMC13348638; doi:10.1186/s13071-026-07441-x)
Supplement: Supplementary file 2 — Supplementary Material 2. [file 13071_2026_7441_MOESM2_ESM.docx]

**Additional file 2**

**Table S1. Definitions of habitat subclass.**

| Subclass | Definition |
| --- | --- |
| Class: Natural | |
| Groundwater pools | Groundwater or rainwater water that accumulates in natural surface depressions |
| Rock holes | Natural depressions that form in rock surfaces due to erosion, weathering or geological processes that collects and retains water |
| Stream margin | Stream edges |
| Tree holes | Natural cavities in trees |
| Plant axils | The angles or junctions between a plant’s stem or branches and its leaves where water can accumulate |
| Class: Man-made from natural materials | |
| Coconut shells | Coconut shells opened by humans that allows water to accumulate |
| Ditches | Channels dug into the ground to manage water flow with a substrate of naturally occurring materials such as soil, sand or gravel |
| Fishponds | Water bodies made by humans for fish cultivation with natural substrates such as soil, sand or gravel |
| Footprints and hoof prints | Water that collects and is retained in ground impressions made by the feet of humans or animals |
| Dug holes | Depressions in the ground created by human activity. |
| Tyre tracks | Ground impressions formed by vehicle wheels that collect and retain water; these habitats are generally ephemeral and rain-dependent, although some may persist for extended periods under suitable environmental conditions. |
| Class: Man-made from artificial materials | |
| Drain | A channel dug in the ground and then covered with concrete or other artificial materials to remove excess water from an area. |
| Large containers (>25l) | with a capacity greater than 25 liters, made of artificial materials such as plastic, metal, glass or concrete. Example: plastic or metal drums, plastic or concrete vessels. |
| Small containers (<10L) | Receptacles or vessels with a capacity less than 10 liters, made of artificial materials such as plastic, metal, glass or concrete. Example: buckets, jerrycans, plastic sheets, cans, pots saucer. |
| Tyres | Circular, rubber structures used on vehicles that when removed from the vehicle discarded or improperly stored can collect and retain rainwater. |
