# Supplementary figures and images for: Larval habitat preferences of Anopheles dirus and Anopheles maculatus in North Sumatra, Indonesia
Source: Parasit Vectors. 2026 May 18;19:286. doi: 10.1186/s13071-026-07441-x (PMC13348638; doi:10.1186/s13071-026-07441-x)

**Additional file 3**


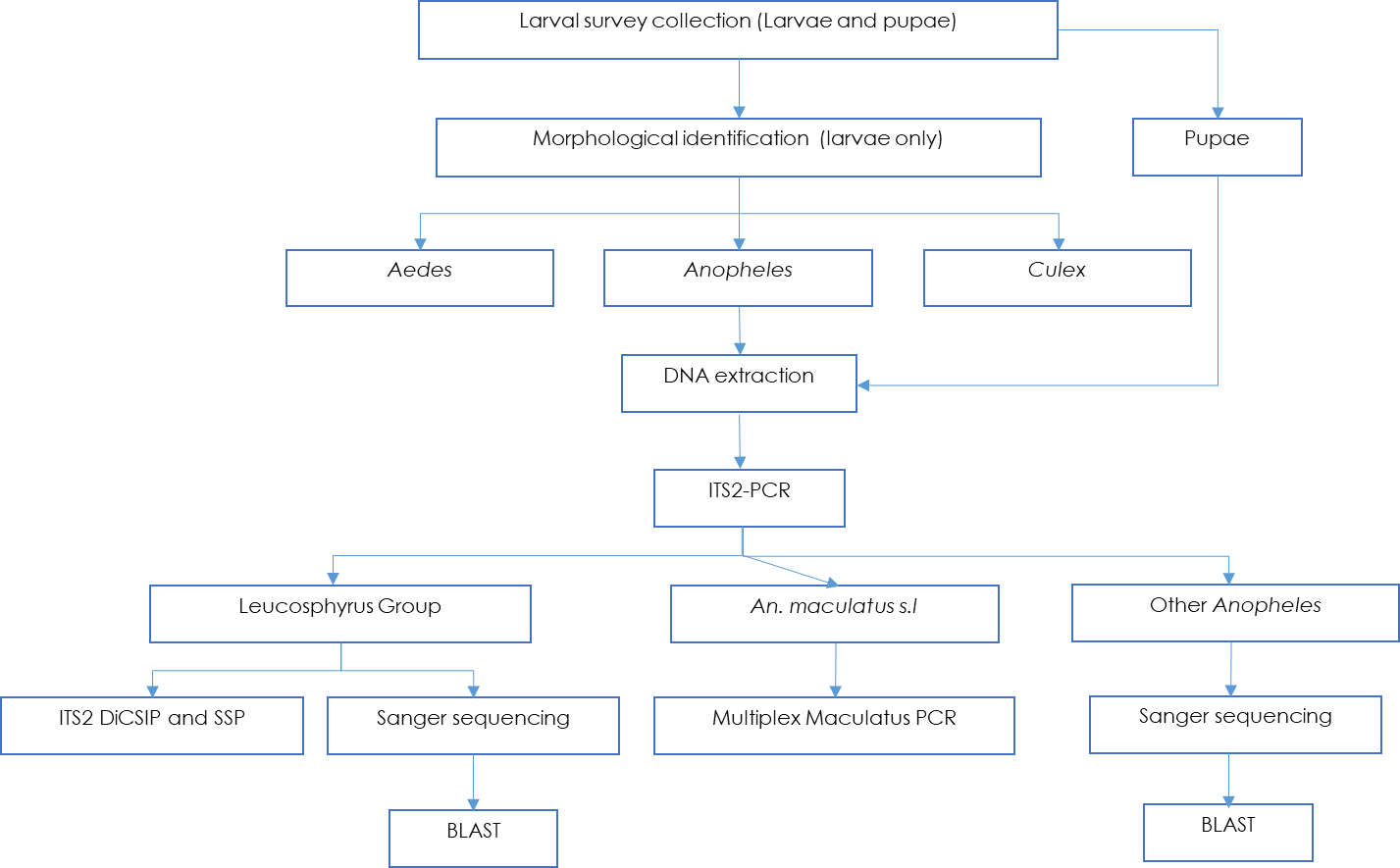


**Fig. S2 Workflow for *Anopheles* identification of larvae and pupae.**

Supplement: Supplementary file 3 — Supplementary Material 3. [file 13071_2026_7441_MOESM3_ESM.docx]

**Additional file 5**


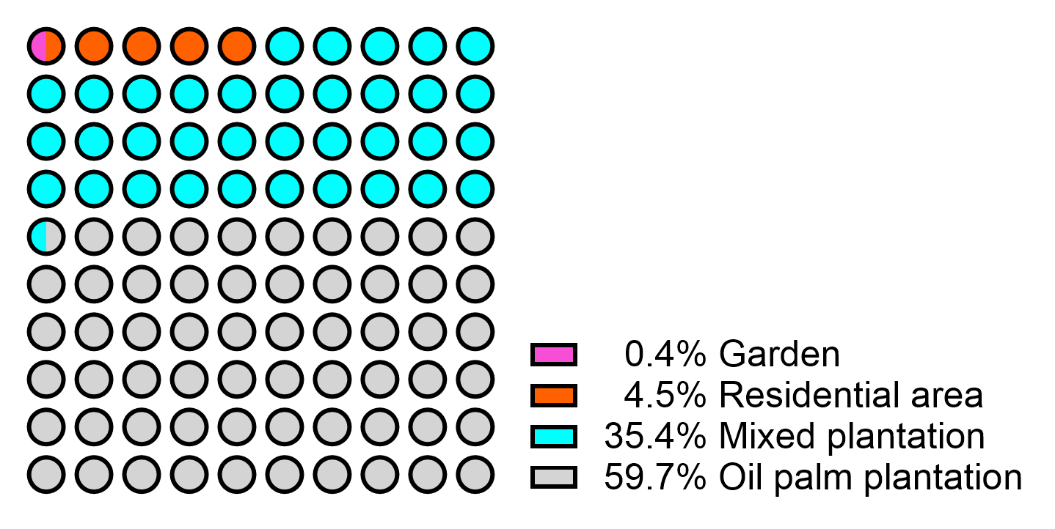


**Fig. S4 Proportion of the area of each surveyed land use type.**

Supplement: Supplementary file 5 — Supplementary Material 5. [file 13071_2026_7441_MOESM5_ESM.docx]
