# Supplementary material for: Larval habitat preferences of Anopheles dirus and Anopheles maculatus in North Sumatra, Indonesia
Source: Parasit Vectors. 2026 May 18;19:286. doi: 10.1186/s13071-026-07441-x (PMC13348638; doi:10.1186/s13071-026-07441-x)
Supplement: Supplementary file 6 — Supplementary Material 6. [file 13071_2026_7441_MOESM6_ESM.docx]

**Additional file 6**

**
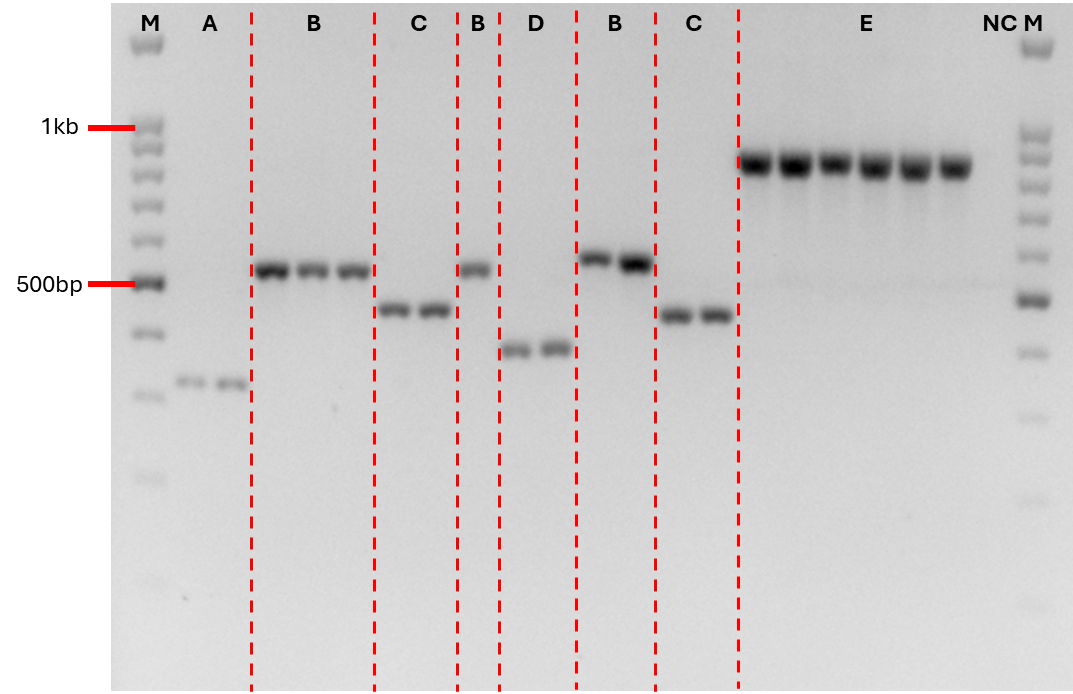
**

**Fig. S5 The ITS2-PCR results of mosquito larvae.** (A) *Armigeres subalbatus*; (B) *Aedes albopictus*; (C) *Anopheles maculatus s.l.* (460bp); (D) *Anopheles kochi;* and (E) Leucosphyrus Group (±880bp), NC: negative control, M = 100 bp ladder marker. Gel was run at 100V for 100 mins.
