# Supplementary material for: Larval habitat preferences of Anopheles dirus and Anopheles maculatus in North Sumatra, Indonesia
Source: Parasit Vectors. 2026 May 18;19:286. doi: 10.1186/s13071-026-07441-x (PMC13348638; doi:10.1186/s13071-026-07441-x)
Supplement: Supplementary file 7 — Supplementary Material 7. [file 13071_2026_7441_MOESM7_ESM.docx]

**Additional file 7**


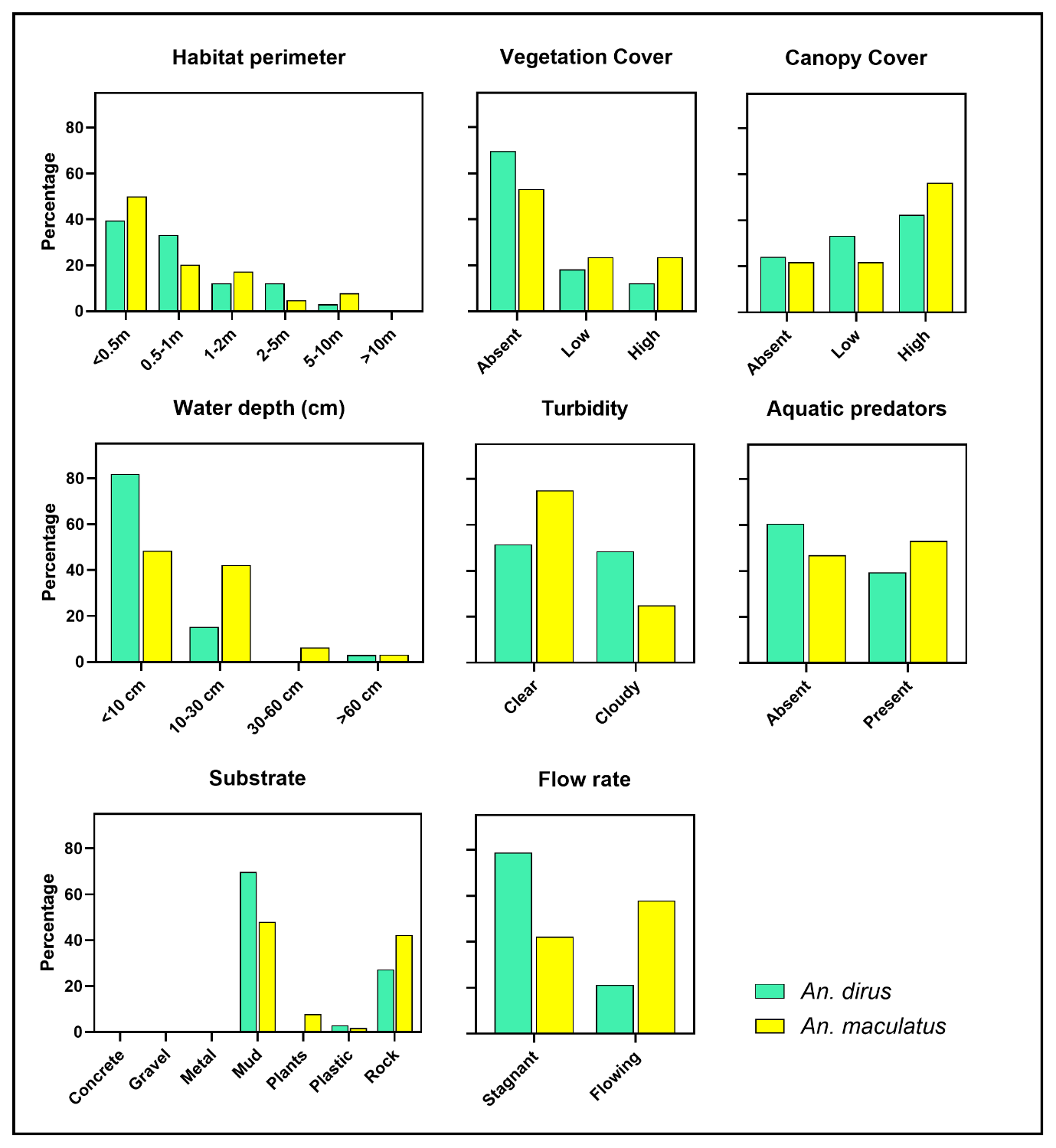


**Fig. S6** **Abiotic and biotic parameters associated with positive *Anopheles* larval habitats.**
