# Supplementary material for: Larval habitat preferences of Anopheles dirus and Anopheles maculatus in North Sumatra, Indonesia
Source: Parasit Vectors. 2026 May 18;19:286. doi: 10.1186/s13071-026-07441-x (PMC13348638; doi:10.1186/s13071-026-07441-x)
Supplement: Supplementary file 8 — Supplementary Material 8. [file 13071_2026_7441_MOESM8_ESM.docx]

**Additional file 8**


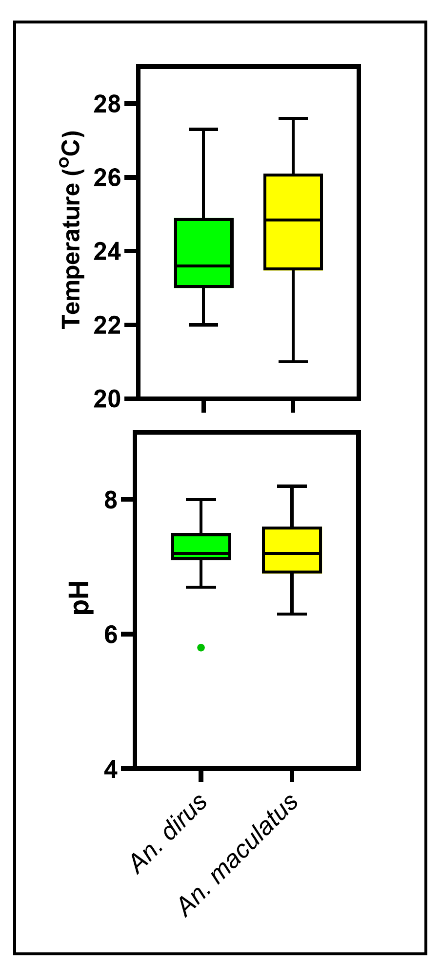


**Fig. S7.** **Associations between water temperature and pH and the presence of *Anopheles* *dirus* and *Anopheles maculatus* larvae in aquatic habitats**.
